# Bidirectional associations between the duration and timing of nocturnal sleep and daytime naps in adolescents differ from weekdays to weekends

**DOI:** 10.1093/sleep/zsae147

**Published:** 2024-06-28

**Authors:** Ruth L F Leong, Liang Tian, Nicole Yu, Teck Boon Teo, Ju Lynn Ong, Michael W L Chee

**Affiliations:** Centre for Sleep and Cognition, Yong Loo Lin School of Medicine, National University of Singapore, Singapore; Centre for Sleep and Cognition, Yong Loo Lin School of Medicine, National University of Singapore, Singapore; Centre for Sleep and Cognition, Yong Loo Lin School of Medicine, National University of Singapore, Singapore; Centre for Sleep and Cognition, Yong Loo Lin School of Medicine, National University of Singapore, Singapore; Centre for Sleep and Cognition, Yong Loo Lin School of Medicine, National University of Singapore, Singapore; Centre for Sleep and Cognition, Yong Loo Lin School of Medicine, National University of Singapore, Singapore

**Keywords:** nap, timing, actigraphy, adolescents

## Abstract

**Study Objectives:**

Previous studies examining bidirectional relationships between nocturnal sleep and napping have focused on sleep duration, leaving a gap in our understanding of how sleep timing contributes. Here, we assessed the duration and timing for night sleep and daytime naps, to evaluate how the previous night’s sleep influences the next day's napping, and how napping influences same-night nocturnal sleep.

**Methods:**

We analyzed sleep diary and actigraphy data from 153 teens (males = 43.8%, mean age = 16.6 years). Participants who never napped were excluded. Nocturnal sleep-nap relationships were investigated using logistic and linear regression models separately for weekdays and weekends.

**Results:**

Participants napped an average of 2.3 times a week. 167 school day naps and 107 weekends were recorded. Naps were on average 82.12 ± 53.34 minutes and the average nap onset was 14:58 ± 3.78 hours. Their duration, start and end times did not significantly differ between weekdays and weekends. Nocturnal sleep duration did not predict next-day nap occurrence or duration. However, on school days, earlier wake times significantly increased the likelihood of napping that day, and advanced nap timing. On weekends, later bedtimes and wake times delayed nap timing. On school days, napping longer than one’s average shortened nocturnal sleep whereas on weekends, waking from a nap later than one’s average delayed bedtimes.

**Conclusions:**

Early wake times increase the likelihood of napping and advance the time of a nap that day. Naps may be detrimental to the same night’s sleep only if they are long and occur late, as these can delay bedtimes and shorten nocturnal sleep duration, especially on school days.

**Clinical trials:**

The Cognitive and Metabolic Effects of Sleep Restriction in Adolescents (NFS4), https://clinicaltrials.gov/study/NCT03333512, ID: NCT03333512. Investigating Preferred Nap Schedules for Adolescents (NFS5), https://clinicaltrials.gov/study/NCT04044885, ID: NCT04044885.

## Graphical Abstract

**Graphical Abstract:**
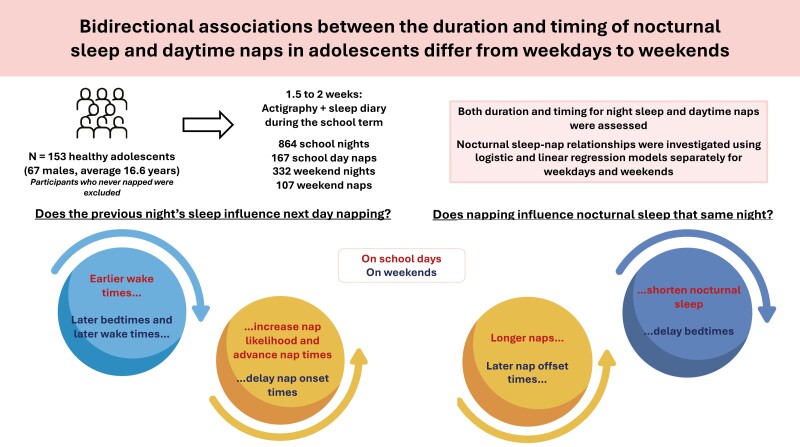

**Statement of Significance**

When nocturnal sleep is chronically insufficient, the opportunity to nap rather than nocturnal sleep duration may determine whether, how long, and when naps are taken the next day. However, there is an interplay between nocturnal sleep and naps, whereby early wake times bring forward the time one naps that day, and where naps that are too long and too late may be detrimental to that night’s sleep, especially for school days where wake times cannot be delayed to extend sleep duration. Appropriate scheduling of the duration and timing of napping should be emphasized when providing sleep hygiene recommendations.

Naps are short periods of sleep that occur outside the main nocturnal sleep period, which can alleviate sleepiness, as well as boost mood and cognition [1, 2]. A meta-analysis of actigraphically measured sleep across 17 countries found that teens aged 13–19 years obtained less than 7 hours of sleep on weekdays and less than 8 hours on weekends [3] – significantly less than what is recommended [4]. In a population-based sample of Brazilian teens, 70% of teens reported “never” or only “sometimes” getting satisfactory sleep at night [5]. With chronic inadequate sleep, the most common reason for napping in teens may be to fulfill sleep requirements and counter symptoms of insufficient nocturnal sleep that impact daily functioning, such as deterioration in performance and fatigue [6].

Surveys suggest that 40% to 58% [5, 7–9] of adolescents ranging from 13 to 19 years report napping at least once a week, with average nap durations reported as 43 minutes in one study [9] and 60 minutes in another [5]. When naps are objectively measured with actigraphy, one study found that up to 89% of adolescents napped at least once a week, with an average duration of 65 minutes [10]. Long naps may indicate a greater need for compensatory sleep, as an Icelandic epidemiological study found that adolescents who were “often sleepy” took longer naps compared to those who were “seldom or never sleepy.” In addition, nappers in their study had 42–53 minutes less sleep at night compared to non-nappers [7]. A closer investigation of the temporal relationships between nocturnal and nap sleep supports this. Measuring sleep with actigraphy across a school week, a study found that in students sleeping an average of 6.5 hours at night, shorter nocturnal sleep predicted a higher likelihood of napping as well as longer naps the next day [10], suggesting that napping in teens may be largely driven by compensatory reasons [11].

Although napping effectively increases the total amount of sleep across 24 hours, the duration and timing of when naps are taken are important considerations, as they influence whether naps have a negative impact on nocturnal sleep. In teens napping an average of 65.5 min (SD = 42.2 minutes), longer naps were associated with later bedtimes and shorter same-night nocturnal sleep duration [10]. However, it is not known what time the naps were taken. Further, how timing may impact nocturnal sleep remains relatively unstudied. When 90-minute naps were scheduled at 15:00 in the afternoon for teens who slept <6.5 hours at night, there was no consistent impact on nocturnal sleep latency. Over five experimental days, only one night showed prolonged sleep latency (approximately 10 min difference) in the nap group compared to the no-nap group [12]. However, an observational study of Japanese teens reported that students who napped later than 17:00, which was common in 35% of their sample, had bedtimes 30 minutes later compared to those who napped between 13:00 and 15:00 [8].

Temporal relationships between the timing of daytime and nighttime sleep have not been examined objectively. In the present investigation, we addressed these gaps by using actigraphy data collected over 1.5 to 2 weeks in teens during the school term to answer the following questions: (1) whether the previous night’s sleep would influence the next day's nap occurrence, nap duration, and timing, (2) whether nap duration and timing would affect that night’s sleep timing and duration. Furthermore, as weekday–weekend differences in schedules during the school term would drive large differences in sleep behavior (as seen in the present data), we did not wish to collapse sleep across the week but rather, wanted to examine weekdays and weekends as separate periods.

## Methods

The present investigation used data from 153 teens collected during the screening periods of each of the Need for Sleep studies [12–16] held between 2015 and 2019 that examined the relationships between schedules of sleep restriction and napping, health, and cognitive outcomes in adolescents. Each of these studies were independently conducted. Participants were recruited from high schools in Singapore and ranged from 15 to 19 years old (mean ± SD age: 16.63 ± 1.12 years, males = 43.8%). As part of the screening procedures, participants’ habitual sleep patterns were recorded for 1.5–2 weeks during which they wore an Actiwatch (Actiwatch-2, Phillips Respironics, Bend, OR) as well as filled in a sleep diary (Table 1). In our Need for Sleep cohorts, approximately 50%–55% of adolescents report napping at least once a week [17]. For the present analyses, only participants who reported taking at least one nap during the entire 1.5–2-week screening period were included in the present investigation. 83.8% of participants from the initial pool met this criterion for inclusion into the present analysis sample.

**Table 1.** Nocturnal Sleep and Nap Parameters by Weekdays and Weekends

	Mean	*SD*	Median
*School nights = 864*			
Bedtime (hh:mm)^a^	00:03	1.30	00:01
Wake time (hh:mm)^a^	07:04	1.47	06:40
Time in bed (min)^a^	420.94	86.74	414.00
Total sleep time (min)^a^	342.07	76.18	330.00
Sleep onset latency (min)	10.83	14.81	6.00
*School day naps = 167*			
Time in bed (min)	79.93	53.82	65.00
Start time (hh:mm)	15:06	3.93	16:00
End time (hh:mm)	16:26	4.22	17:38
*Weekend nights = 332*			
Bedtime (hh:mm)^a^	00:43	1.43	00:36
Wake time (hh:mm)^a^	08:47	1.60	08:44
Time in bed (min)^a^	483.54	94.97	480.00
Total sleep time (min)^a^	398.36	89.48	392.00
Sleep onset latency (min)	10.53	13.43	6.00
*Weekend naps = 107*			
Time in bed (min)	82.00	51.39	68.00
Start time (hh:mm)	15:07	3.00	15:26
End time (hh:mm)	16:29	3.07	16:50

School nights (next day is a school day): Sunday, Monday, Tuesday, Wednesday, and Thursday.

SD, standard deviation.

^a^Denotes significant main effect of school day/weekend.

In the sleep diary, for each day, participants recorded their bedtime, wake time, and sleep latency for nocturnal sleep periods, as well as the start and end time of any nap taken. Notably, naps were distinguished from nocturnal sleep in the sleep diary with separate columns provided for the recording of the two types of sleep entries. For each day, they also indicated whether the next day was a school day or not. Using this, we were able to confirm if weekdays were indeed school days, and weekends were indeed non-school days. If there were unusual patterns (e.g. participant reported a non-school day despite it being a weekday, yet no indication of a national or school holiday), these days were removed as we were not able to re-contact participants to confirm the details of these days. We also excluded public school holidays based on the national academic calendar for each of the specific years we recruited participants. In the present study, school nights refer to nights before a school day, (i.e. Sunday night to Thursday night), and weekend nights refer to nights before a non-school day (i.e. Friday night and Saturday night).

Although naps have different definitions depending on the focus of the investigation [18], in our study naps are defined as a period of sleep distinct from a main nocturnal period. We focus on “daytime” naps occurring during the period between 05:00 and 23:59. Although there has been no consensus on the minimum or maximum duration required for a nap to be considered a nap, we wished to avoid situations of polyphasic sleep where one may intentionally divide a desired sleep period into two more or less equal bouts. As we sought to investigate nap-night sleep relationships in the context of regular school-term schedules, we also looked to exclude anomalous days which may have occurred due to exceptional reasons. Hence, we excluded nap periods exceeding 240 minutes as these were deemed to be unusually long in the context of the school term and may have occurred due to abnormal programming that day (e.g. sickness, or a school absence for other reasons).

These studies were approved by the Institutional Review Board of the National University of Singapore and informed consent was obtained from both participants and their legal guardians.

### Actigraphy

Data were collected in 2-minute epochs and scored using the Actiware 6.2 software. Sleep parameters such as time in bed (TIB), total sleep time (TST), sleep onset latency (SOL), bedtime, and waketime, were calculated using the software algorithm set to medium sensitivity (wake events defined as having an activity count of ≥40). While TIB is calculated as the interval from bedtime to waketime, TST is computed by summing all sleep epochs within this interval.

Sleep and wake times were determined based on the participant’s self-reported timings recorded in a sleep diary, as well as event markers on the actogram. We manually inspected the actigraphy data with reference to the sleep diary data and event markers on the actogram, and hand-scored the rest intervals from a combination of the event marker and diary, with preference given to the event marker. Where needed, the actogram was adjusted before generating sleep statistics. If a nap was reported but there was no evidence of a nap by actigraphy, the data was not included. Only naps with a discrepancy of 30 minutes and below between sleep diary and actigraphy-recorded start and end times were included in the analyses. For entries with discrepancy >30 minutes, we excluded the entry from analyses. If necessary, changes in light and activity levels were referred to for defining the sleep period.

### Questionnaires

Participants also completed questionnaires assessing their non-verbal reasoning abilities, sleep quality, habitual sleep patterns, daytime sleepiness, mood, and caffeine consumption (Table 2). The scores on these questionnaires are reported for characterization of the sample, and are not directly involved in the present analyses. The Raven’s Progressive Matrices [19] were used to measure non-verbal reasoning. Sleep quality was assessed by the Pittsburgh Sleep Quality Index [20] and symptoms of sleep restriction were assessed with the Chronic Sleep Reduction Questionnaire [21]. Morningness–eveningess preference was measured with the Morningness-eveningness questionnaire [22]. Levels of daytime sleepiness were assessed with the Epworth Sleepiness Scale [23]. Symptoms of anxiety and depression were measured with the Beck Anxiety Inventory [24] and Beck Depression Inventory [25] respectively. Participants were also asked how many cups of caffeinated drinks they consumed in a day.

**Table 2.** Sample Characteristics

	Mean/frequency	*SD*
*n*	153	—
Age (y)	16.63	1.12
Gender (no. of males)	67	—
Days of data recorded	8.68	3.68
Average naps per week	2.30	2.17
Ravens Progressive Matrices Score	8.98	1.87
PSQI global score	5.38	2.22
CSRQ subscale: shortness of Sleep	13.22	2.21
CSRQ subscale: irritation	6.80	1.94
CSRQ subscale: energy Loss	8.33	2.06
CSRQ subscale: sleepiness	8.11	1.86
Epworth Sleepiness Score	7.97	3.42
Berlin questionnaire	0.03	0.16
Beck’s anxiety inventory	0.94	0.86
Beck’s depression inventory	0.91	1.20
Caffeine (cups/day)	0.60	0.79

SD, standard deviation; PSQI, Pittsburgh Sleep Quality Index; CSRQ, Chronic Sleep Reduction Questionnaire.

### Statistical methods

Data were analyzed in RStudio using the *lme4*, *lmer,* and *glmnet* packages.

To minimize discrepancies in the data, nights where TIB was <4 hours were not included in the analyses [26]. Such nights were treated as idiosyncratic days of severe sleep deprivation. As we wished to examine habitual sleep patterns, we wanted to exclude these exceptional nights. Days that were indicated as school days, but had wake times recorded later than 08:00, were also not included as the majority of schools in Singapore start before 08:00. Daytime sleep periods that exceeded 240 minutes, or bouts of nap sleep that occurred between 00:00 and 04:59, were also excluded from all analyses as we wanted to limit our investigation to daytime naps [27]. We excluded 12 naps that exceeded 240 minutes and 4 naps that occurred between 00:00 and 04:59.

Wilcoxon signed-rank tests were used to test for significant differences between school day and weekend nocturnal sleep and nap duration and timing. Relationships between nocturnal sleep parameters (duration [TIB and TST], bedtime, wake time, SOL) and nap sleep parameters (nap duration [TIB], nap start time, nap end time) were investigated using multilevel logistic (where nap occurrence was the dependent variable) and linear regression models controlling for age and gender. The sleep predictors were entered into separate models to reduce the potential for collinearity. We also checked for collinearity by examining the variance inflation factor. We found no significant collinearity (variance inflation factor below 4, and tolerance below 0.25). Random intercepts and random slopes (where convergence was possible) were included in all models to account for possible interindividual differences in associations between sleep and outcome variables at the day-to-day level. Nocturnal sleep and nap sleep parameters were treated as continuous variables. Multilevel logistic regression models were used to examine whether the previous night’s sleep parameters would predict whether or not a nap occurred the next day (nap = 1, no nap = 0). For days where a nap occurred, multilevel linear regression models were used to examine whether the previous night’s sleep parameters influenced the nap sleep parameters, as well as whether nap sleep parameters influenced sleep the same night. For time points where naps did not occur, nap duration was input as 0, and nap timing was input as missing.

Given the different schedules of time use during the school week versus weekends, we elected not to collapse the sleep data but instead, examined nocturnal-nap sleep relationships separately for weekdays and weekends. However, models testing for moderation within which a day factor and an interaction term (day × sleep/nap variable) were included, were also analyzed. These results may be found in Supplementary Material.

## Results

### Habitual nocturnal sleep and napping

Only participants who reported taking at least one nap during the screening period were included in the present analysis (see methods). A total of 153 participants (67 males) aged on average 16.6 years were included in the analyses and each individual provided an average of 9.6 days of data (Table 2). Participants had an average PSQI global score of 5.38 (SD = 2.22) and scored an average of 7.97 on the ESS. CSRQ scores suggest that participants did not exceed the clinical threshold of chronic sleep reduction. BAI and BDI scores suggest minimal clinical symptoms of anxiety and depression. Participants reported consuming 0.6 cups of caffeine a day.

Compared to school days, on weekends, adolescents went to bed 40 minutes later (*p* < .001) but also woke 100 minutes later (*p *< .001), thus obtaining an average of 60 minutes more sleep on the weekend (*ps* < .001). Average sleep duration (TST) on school days was 5.70 hours (SD = 1.27) compared to 6.64 hours (SD = 1.49) on weekends.

**Figure 1.**
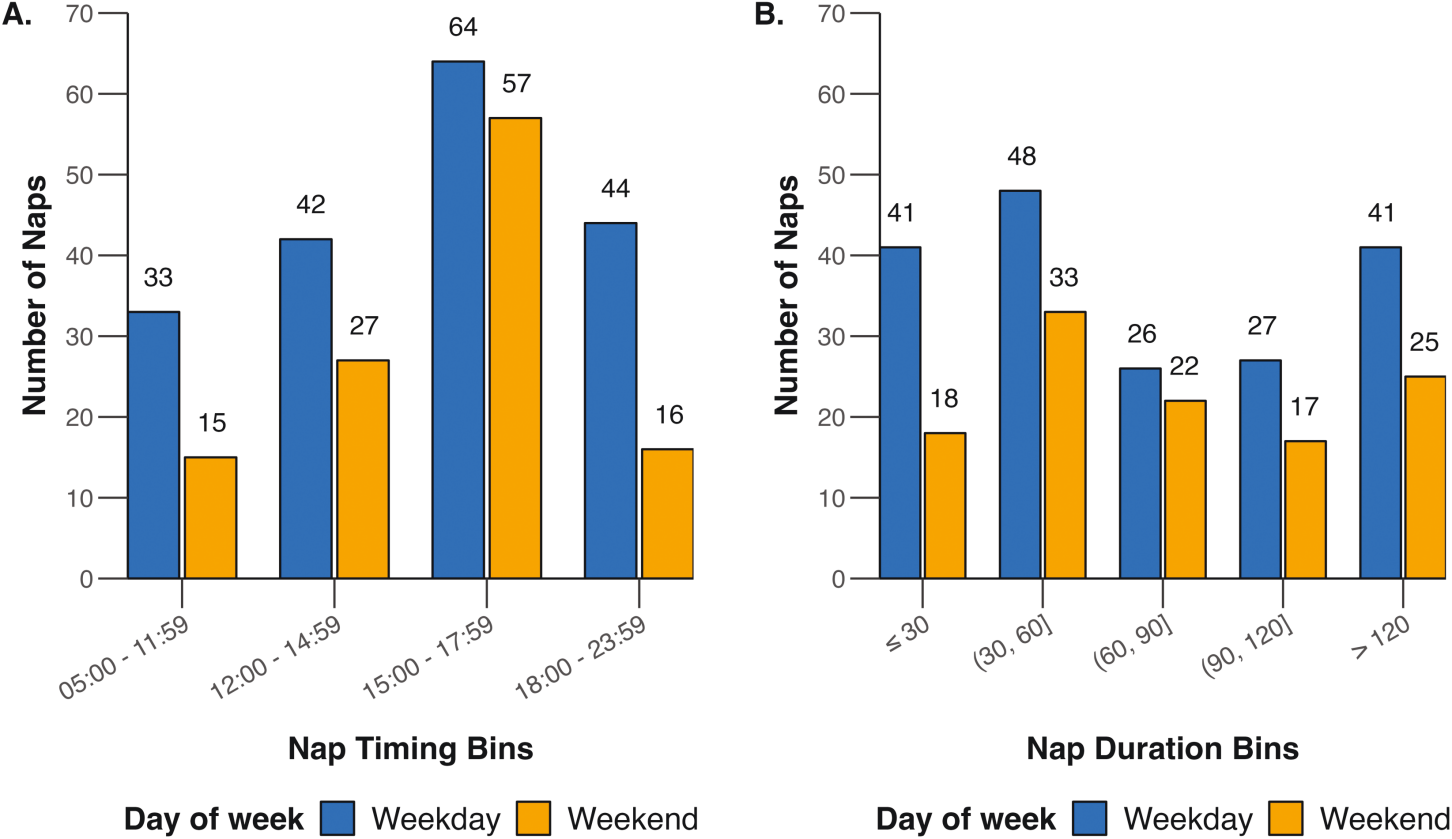
Histogram depicting (A) nap start times and (B) nap durations on weekdays and weekends.

Participants took an average of 2.3 naps a week, and the likelihood of napping on a school day was 19.3%, and 32.2% on a non-school day. Regardless of the day of the week, actigraphy-defined naps were 82.12 ± 53.34 minutes long and the average nap onset was 14:58 ± 3.78 hours in the afternoon. There were no significant differences in nap duration (*p* = .41), or nap start times (onset) and end times (offset) between weekdays and weekends (*ps* > .38). To illustrate the distribution of nap start times in the sample, histograms were plotted according to the following categories: early morning (05:00–11:59), early afternoon (12:00–14:59), late afternoon (15:00–17:59), and evening (18:00–23:59; Figure 1A). On both weekdays and weekends, naps were most commonly taken between 15:00 and 18:00. Nap durations were categorized into 30-minute bins (Figure 1B). Naps ranged from less than 30 to 120 minutes or longer, with the most common duration of naps being between 30 and 60 minutes, followed by those longer than 120 minutes.

### Effects of the previous night’s sleep on next-day nap occurrence, duration, and timing

On school days, earlier wake times significantly increased the likelihood of napping that day (B = −0.42, SE = 0.16, *p* = .008), and were also associated with earlier nap start times (B = 1.33, SE = 0.53, *p *= .014) and nap wake times that day (B = 1.51, SE = 0.56, *p* = .008; Table 3). Specifically, waking 1 hour earlier than the sample average brought nap start and end timings forward by 78 and 91 minutes, respectively. However, nocturnal duration or bedtime did not significantly predict whether a nap would be taken, or what duration or timing of the nap the next day would be (*ps* > .053). There were no significant within-participant effects (*ps *> .060).

**Table 3.** Unstandardized Beta (Standard Error) Values for the Effects of the Previous Night’s Sleep Parameters on the Next Day’s Nap Parameters

	Nap TIB (min)	Nap start time (h)	Nap end time (h)	Nap occurrence
Weekday (no. of observations = 436)				
*Between-person effects*				
Night TIB (min)	0.036 (0.09)	0.009 (0.01)	0.009 (0.01)	−0.003 (<0.01)
Night TST (min)	0.096 (0.11)	0.016 (0.01)	0.016 (0.01)	<−0.001 (<0.01)
Night SOL (min)	−0.321 (0.47)	−0.014 (0.04)	−0.020 (0.04)	0.006 (0.01)
Bedtime (h)	8.913 (4.68)	0.296 (0.37)	0.443 (0.40)	0.038 (0.09)
Wake time (h)	13.325 (6.84)	1.328 (0.53) *	1.509 (0.56) **	−0.420 (0.16) **
*Within-person effects*				
Night TIB (min)	−0.133 (0.07)	0.003 (<0.01)	0.001 (0.01)	—
Night TST (min)	−0.101 (0.07)	0.004 (<0.01)	0.004 (<0.01)	—
Night SOL (min)	−0.016 (0.28)	−0.001 (0.02)	− 0.001 (0.02)	—
Bedtime (h)	2.436 (4.96)	−0.304 (0.33)	−0.266 (0.34)	—
Wake time (h)	0.193 (6.17)	0.641 (0.46)	0.584 (0.47)	—
Weekend (no. of observations = 152)				
*Between-person effects*				
Night TIB (min)	0.009 (0.13)	0.010 (0.01)	0.009 (0.01)	−0.002 (<0.01)
Night TST (min)	−0.031 (0.14)	0.002 (0.01)	0.001 (0.01)	0.001 (<0.01)
Night SOL (min)	−0.056 (0.59)	0.066 (0.04)	0.064 (0.04)	−0.014 (0.01)
Bedtime (h)	3.479 (5.79)	0.803 (0.38) *	0.896 (0.39) *	−0.035 (0.12)
Wake time (h)	3.360 (5.58)	1.075 (0.33) **	1.149 (0.34) **	−0.159 (0.11)
*Within-person effects*				
Night TIB (min)	−0.054 (0.06)	0.009 (<0.01) *	0.010 (<0.01) *	—
Night TST (min)	−0.051 (0.07)	0.012 (<0.01) **	0.013 (<0.01) **	—
Night SOL (min)	−0.117 (0.59)	0.012 (0.04)	0.010 (0.04)	—
Bedtime (h)	−2.931 (4.72)	0.417 (0.29)	0.322 (0.29)	—
Wake time (h)	−5.957 (4.13)	0.966 (0.24) ***	0.875 (0.24) ***	—

Models included age and gender as covariates.

TIB, time in bed; TST, total sleep time; SOL, sleep onset latency.

*p < .05, **p < .01, ***p < .001.

On weekends, at the between-participant level, later bedtimes and later wake times were significantly associated with later nap times. Specifically, going to bed 1 h later than the sample average delayed nap times by 48 minutes the next day (B = 0.80, SE = 0.38, *p *= .040), and waking 1 hour later delayed naps by 65 minutes (B = 1.08, SE = 0.33, *p* = .002). At the within-participant level, waking 1 hour later than one’s average wake time was associated with napping 1 hour later than one’s usual nap time (B = 0.97, SE = 0.24, *p *< .001). Sleeping longer than one’s average duration (TIB and TST) was also significantly associated with napping later than one’s usual nap time, but these delays were in the range of under a minute (Bs = 0.01, SEs < 0.01, *ps* = .004–.019).

### Effects of daytime nap duration and timing on the same night's sleep

Next, we asked whether naps would affect nocturnal sleep that same night. On school days, at the between-participant level, napping for 1 hour longer shortened nocturnal sleep (TIB) by 22 minutes (B = −0.37, SE = 0.13, *p* = .004, Table 4); the delay in bedtimes were significant but effects were comparatively small (B = 0.01, SE < 0.01, *p* < .001). At the within-participant level, napping 1 hour longer than one’s own average nap duration was also associated with 29 minutes of shorter sleep (TST) than one’s own average nocturnal duration (B = −0.48, SE = 0.17, *p* = .004). Similarly, this also significantly delayed one’s bedtimes but to a smaller extent (B = 0.01, SE < 0.01, *p *= .005). SOL was not significantly affected (*p *> .08). There were no significant effects of nap timing on nocturnal sleep parameters.

**Table 4.** Unstandardized Beta (Standard Error) Values for the Effects of Daytime Nap Parameters on the Subsequent Night’s Sleep Parameters

Nocturnal sleep parameters				
	TIB (min)	TST (min)	SOL (min)	Bedtime (h)
Weekday (no. of observations = 138)				
*Between-person effects*				
Nap TIB (mins)	−0.373 (0.13) **	−0.257 (0.13)	0.057 (0.03)	0.008 (<0.01) ***
Nap start time (h)	−0.517 (1.50)	0.139 (1.51)	0.365 (0.42)	0.031 (0.03)
Nap end time (h)	−0.986 (1.39)	−0.182 (1.41)	0.442 (0.39)	0.039 (0.02)
*Within-person effects*				
Nap TIB (min)	−0.415 (0.17)*	−0.481 (0.17) **	0.057 (0.03)	0.008 (<0.01) **
Nap start time (h)	−2.862 (3.16)	−0.243 (3.11)	−0.988 (0.59)	0.066 (0.05)
Nap end time (h)	−4.503 (3.05)	−2.454 (3.02)	−0.663 (0.57)	0.095 (0.05)
Weekend (no. of observations = 57)				
*Between-person effects*				
Nap TIB (min)	−0.414 (0.25)	−0.370 (0.25)	0.041 (0.04)	0.010 (<0.01)
Nap start time (h)	9.010 (6.01)	6.700 (6.06)	1.762 (0.88)	0.214 (0.11)
Nap end time (h)	4.282 (5.70)	2.697 (5.71)	1.931 (0.81) *	0.268 (0.10) *
*Within-person effects*				
Nap TIB (min)	−0.029 (0.41)	−0.001 (0.42)	0.024 (0.06)	−0.003 (0.01)
Nap start time (h)	−2.810 (6.24)	−3.797 (6.28)	0.747 (0.94)	0.235 (0.05) **
Nap end time (h)	−5.560 (6.35)	−6.116 (6.36)	0.882 (0.93)	0.260 (0.06) **

Models included age and gender as covariates.

TIB, time in bed; TST, total sleep time; SOL, sleep onset latency.

*p < .05, **p < .01, ***p < .001.

However, on weekends, at the between-participant level, waking from a nap 1 hour later increased the time taken to fall asleep by 2 minutes (B = 1.93, SE = 0.81, *p *= .020), and delayed bedtimes by 16 minutes (B = 0.27, SE = 0.10, *p* = .011). At the within-participant level, waking from a nap 1 hour later than one’s own average time delayed bedtimes 16 minutes beyond one’s own average bedtime (B = 0.26, SE = 0.06, *p *= .001). However, this did not affect sleep duration (*ps* > .340). There were no significant effects of nap duration on nocturnal sleep parameters.

## Discussion

Our findings examined napping in the context of inadequate nocturnal sleep. This is reflected in both the objectively measured short sleep at night (school days: 5.70 hours; weekends: 6.64 hours) as well as the generally long length of naps taken (median nap duration: 65–68 minutes). Notably, our sample was made up of nappers, as only those who reported napping at least once during the entire screening period were included in the analyses. In our sample, nocturnal duration did not predict whether a nap was taken, whereas a previous study on teens had shown that shorter nocturnal sleep predicted nap occurrence the next day [10]. Sleeping comparatively less at an average of <6 hours nightly, likely raises nap propensity. However, the opportunity to nap may have been limited by scheduling constraints. Nonetheless, early wake times did predict higher nap occurrence for that day and advanced nap timing.

With local school start times already early (07:30 am in Singapore), students must wake before their natural biological rise time. Waking earlier than average may lead to significant sleepiness by midday and a higher drive to nap that day. With a longer period of wakefulness, the buildup of sleep pressure would have accumulated sooner, giving rise to an earlier-than-usual nap time. This shift in nap times in accordance with wake times was also seen on weekends. On weekends, waking on average later (between-participant) and waking later than one’s own average (within-participant) shifted nap timings later, as the buildup of sleep pressure accumulated later than average that day.

The second question we asked was whether napping would influence nocturnal sleep that same night. In our sample, we observed long and late naps, with many having naps that were 2 hours or longer, and many waking from these long naps at almost evening time. We found that on school days, these long naps delayed bedtimes and shortened nocturnal sleep that night, while on weekends, the late naps delayed bedtimes but did not curtail sleep duration. On school days, wake times are constrained by early school start times, and teens have limited opportunities to extend sleep duration by waking later after staying up late. On weekends, although bedtimes are later, teens have the flexibility to wake later and extend sleep duration to compensate for late bedtimes.

Compared to adults, teens appear to nap for a longer duration. While Brazilian [5], American [10], and Singaporean teens [17] nap for an average of 60–84 minutes, adults (age range: 25 to 55 years) in the United States, Canada, Mexico, United Kingdom, Germany, and Japan report taking naps in the range of 33 to 45 mins [28]. Notably, naps in university students are also long, ranging from 75 [29] to 81 minutes [30] in US samples, and 62 minutes in a Singaporean sample [31]. This may point to the greater need in adolescents and younger adults to fulfill higher sleep requirements across the 24-hour period compared to older adults.

Many guidelines recommend naps of around 30–45 minutes, in line with recent evidence that a nap containing 30 minutes of actual sleep, provisioning 10 minutes to fall asleep, has benefits to alertness, performance, and mood, and may have the best tradeoff between practicability and benefit [1]. Although sleep and memory experimental studies often use naps of 90 to 120 minutes to test mechanistic contributions of NREM and REM sleep, several studies suggest that a 30-minute nap already provides functional benefits to memory consolidation and encoding [1, 2, 32]. While excessively long naps have always been discouraged, the reasons for this have been related to the association with greater sleep inertia upon waking [18]. However, this concern may apply more to naps taken after major sleep deprivation rather than after a normal night of sleep [33]. Moreover, the dip in alertness along with performance decrements due to sleep inertia tends to be most prominent at 5 minutes post-nap and often fully dissipates within 20–30 minutes or less [1, 34–36]. Our findings suggest that the more compelling reason to discourage long naps may be because of their potential to delay bedtimes and curtail sleep duration that night, especially for school-going teens who have fixed early wake times the next day and cannot compensate for delayed bedtimes. In college students, longer nap durations were associated with a delay in bedtimes but not shorter nocturnal sleep duration [29, 30], perhaps due to having greater flexibility than school-going teens to wake later thereby extending sleep duration.

Our findings suggest that patterns of napping may reflect the adequacy of sleep at night and are dependent on the opportunity to nap as well as the flexibility to adjust wake times. While naps have clear benefits to cognitive performance [2] and can effectively counter the negative consequences of inadequate sleep at night [37], the timely scheduling of naps should be emphasized when providing sleep hygiene recommendations so as not to inadvertently compromise nocturnal sleep. However, it should also be noted that factors linked to long naps may delay bedtimes in a manner not unrelated to naps, for example, napping in anticipation of an intentional late night of studying, much like prophylactic napping before a night shift [38]. Delaying bedtimes due to the pressure to complete schoolwork is prevalent among students in competitive academic settings [39]. Indeed, this cultural factor may drive the way that sleep is distributed in this group, with comparatively short nocturnal sleep [40] and relatively longer naps in the daytime (average nap duration of 82 mins in the present sample versus 65 minutes reported in Jakubowski et al. [10]). Future studies examining nocturnal-nap sleep relationships should include reasons for naps in the sleep diary log.

## Limitations

The present sample of teens were recruited from a tier of high-performing schools in Singapore and may experience greater academic pressures than their peers in other samples. Strict academic schedules and relatively short nocturnal sleep in this group may limit the generalizability of findings, and the sociocultural environment should be taken into consideration when comparing teen sleep and nap patterns in different settings.

Compared to polysomnography, actigraphy is known to overestimate TST in adults [41, 42], but underestimates sleep in adolescents possibly because of greater movement during sleep in the younger group [15, 43, 44]. This underestimation of TST compared to PSG in our cohort of teens has been documented in a previous investigation [45]. Nonetheless, actigraphy is still generally regarded as a reliable measure of naps [46, 47], particularly in a healthy sample and when used alongside sleep diary records [10], as in the present study.

## Conclusions

When nocturnal sleep is chronically insufficient, the opportunity to nap rather than nocturnal sleep duration determines whether, how long, and when naps are taken the next day. Early wake times increase the likelihood of napping that day and also bring forward nap timing. Very long and late naps can delay bedtimes and shorten sleep duration at night, so the appropriate scheduling of the duration and timing of napping are required to avoid disrupting nocturnal sleep.

## Supplementary material

Supplementary material is available at *SLEEP* online.

zsae147_suppl_Supplementary_Materials

## References

[1] Leong RLF , Lau T, Dicom AR, Teo TB, Ong JL, Chee MWL. Influence of mid-afternoon nap duration and sleep parameters on memory encoding, mood, processing speed, and vigilance. Sleep. 2023;46(4). doi: 10.1093/sleep/zsad025 PMC10091091 36775965

[2] Leong RLF , Lo JC, Chee MWL. Systematic review and meta-analyses on the effects of afternoon napping on cognition. Sleep Med Rev. 2022;65:101666. doi: 10.1016/j.smrv.2022.101666 36041284

[3] Galland BC , Short MA, Terrill P, et al. Establishing normal values for pediatric nighttime sleep measured by actigraphy: a systematic review and meta-analysis. Sleep. 2018;41(4). doi: 10.1093/sleep/zsy017 29590464

[4] Hirshkowitz M , Whiton K, Albert SM, et al. National Sleep Foundation’s updated sleep duration recommendations: final report. Sleep Health. 2015;1(4):233–243. doi: 10.1016/j.sleh.2015.10.004 29073398

[5] Santos JS , Pereira SIR, Louzada FM. Chronic sleep restriction triggers inadequate napping habits in adolescents: a population-based study. Sleep Med. 2021;83:115–122. doi: 10.1016/j.sleep.2021.04.016 33991891

[6] Short MA , Chee MWL. Adolescent sleep restriction effects on cognition and mood. Prog Brain Res. 2019;246:55–71. doi: 10.1016/bs.pbr.2019.02.008 31072563

[7] Thorleifsdottir B , Bjornsson JK, Benediktsdottir B, Gislason T, Kristbjarnarson H. Sleep and sleep habits from childhood to young adulthood over a 10-year period. J Psychosom Res. 2002;53(1):529–537. doi: 10.1016/s0022-3999(02)00444-0 12127168

[8] Fukuda K , Ishihara K. Routine evening naps and night-time sleep patterns in junior high and high school students. Psychiatry Clin Neurosci. 2002;56(3):229–230. doi: 10.1046/j.1440-1819.2002.00986.x 12047570

[9] Foundation NS. 2011 Sleep in America Poll: Communications Technology and Sleep. Washington (DC): National Sleep Foundation; 2011.

[10] Jakubowski KP , Hall MH, Lee L, Matthews KA. Temporal relationships between napping and nocturnal sleep in healthy adolescents. Behav Sleep Med. 2017;15(4):257–269. doi: 10.1080/15402002.2015.1126595 27078714 PMC6499385

[11] Gradisar M , Wright H, Robinson J, Paine S, Gamble A. Adolescent napping behavior: Comparisons of school week versus weekend sleep patterns. Sleep Biol Rhythms. 2008;6:183–186. doi: 10.1111/j.1479-8425.2008.00351.x

[12] Lo JC , Leong RLF, Ng ASC, et al. Cognitive effects of split and continuous sleep schedules in adolescents differ according to total sleep opportunity. Sleep. 2020;43(12). doi: 10.1093/sleep/zsaa129 PMC8061132 32619240

[13] Cousins JN , Sasmita K, Chee MWL. Memory encoding is impaired after multiple nights of partial sleep restriction. J Sleep Res. 2018;27(1):138–145. doi: 10.1111/jsr.12578 28677325

[14] Lo JC , Lee SM, Teo LM, Lim J, Gooley JJ, Chee MW. Neurobehavioral impact of successive cycles of sleep restriction with and without naps in adolescents. Sleep. 2017;40(2). doi: 10.1093/sleep/zsw042 PMC5806570 28364507

[15] Lo JC , Ong JL, Leong RL, Gooley JJ, Chee MW. Cognitive performance, sleepiness, and mood in partially sleep deprived adolescents: The Need for Sleep Study. Sleep. 2016;39(3):687–698. doi: 10.5665/sleep.5552 26612392 PMC4763363

[16] Lo JC , Twan DCK, Karamchedu S, et al. Differential effects of split and continuous sleep on neurobehavioral function and glucose tolerance in sleep-restricted adolescents. Sleep. 2019;42(5). doi: 10.1093/sleep/zsz037 PMC6519912 30753648

[17] Leong RLF , Yu N, Ong JL, et al. Memory performance following napping in habitual and non-habitual nappers. Sleep. 2021;44(6). doi: 10.1093/sleep/zsab038 PMC8193563 33313925

[18] Milner CE , Cote KA. Benefits of napping in healthy adults: impact of nap length, time of day, age, and experience with napping. J Sleep Res. 2009;18(2):272–281. doi: 10.1111/j.1365-2869.2008.00718.x 19645971

[19] Raven J. Advanced Progressive Matrices: Set II. (1962 Revision). London: H. K. Lewis.; 1978.

[20] Buysse DJ , Reynolds CF, 3rd, Monk TH, Berman SR, Kupfer DJ. The Pittsburgh Sleep Quality Index: a new instrument for psychiatric practice and research. Psychiatry Res. 1989;28(2):193–213. doi: 10.1016/0165-1781(89)90047-4 2748771

[21] Meijer AM. Chronic sleep reduction, functioning at school and school achievement in preadolescents. J Sleep Res. 2008;17(4):395–405. doi: 10.1111/j.1365-2869.2008.00677.x 19021856

[22] Horne JA , Ostberg O. A self-assessment questionnaire to determine morningness-eveningness in human circadian rhythms. Int J Chronobiol. 1976;4(2):97–110. 1027738

[23] Johns MWA. A new method for measuring daytime sleepiness: the Epworth sleepiness scale. Sleep. 1991;14(6):540–545. 1798888 10.1093/sleep/14.6.540

[24] Beck AT , Epstein N, Brown G, Steer R. Beck Anxiety Inventory. San Antonio, TX: APA PsycTests; 1988.

[25] Beck AT , Ward CH, Mendelson M, Mock J, Erbauch J. Beck Depression Inventory (BDI). San Antonio, TX: APA PsycTests; 1961.

[26] Hausler N , Marques-Vidal P, Haba-Rubio J, Heinzer R. Does sleep predict next-day napping or does napping influence same-day nocturnal sleep? Results of a population-based ecological momentary assessment study. Sleep Med. 2019;61:31–36. doi: 10.1016/j.sleep.2019.04.014 31300205

[27] Ye L , Hutton Johnson S, Keane K, Manasia M, Gregas M. Napping in college students and its relationship with nighttime sleep. J Am Coll Health. 2015;63(2):88–97. doi: 10.1080/07448481.2014.983926 25397662

[28] Foundation NS. International Bedroom Poll. Washington (DC): National Sleep Foundation; 2013.

[29] Mead MP , Huynh P, Le TQ, Irish LA. Temporal Associations between daytime napping and nocturnal sleep: an exploration of random slopes. Ann Behav Med. 2022;56(11):1101–1109. doi: 10.1093/abm/kaac006 35195690 PMC9923793

[30] Rea EM , Nicholson LM, Mead MP, Egbert AH, Bohnert AM. Daily relations between nap occurrence, duration, and timing and nocturnal sleep patterns in college students. Sleep Health. 2022;8(4):356–363. doi: 10.1016/j.sleh.2022.05.002 35732554 PMC9378669

[31] Ng ASC , Massar SAA, Bei B, Chee MWL. Assessing “readiness” by tracking fluctuations in daily sleep duration and their effects on daily mood, motivation, and sleepiness. Sleep Med. 2023;112:30–38. doi: 10.1016/j.sleep.2023.09.028 37804715

[32] Wang SY , Baker KC, Culbreth JL, et al. “Sleep-dependent” memory consolidation? Brief periods of post-training rest and sleep provide an equivalent benefit for both declarative and procedural memory. Learn Mem. 2021;28(6):195–203. doi: 10.1101/lm.053330.120 34011516 PMC8139635

[33] Dinges D , Orne M, Orne E. Sleep depth and other factors associated with performance upon abrupt awakening. Sleep Res. 1985;14:92.

[34] McDevitt EA , Sattari N, Duggan KA, et al. The impact of frequent napping and nap practice on sleep-dependent memory in humans. Sci Rep. 2018;8(1):15053. doi: 10.1038/s41598-018-33209-0 30305652 PMC6180010

[35] Brooks A , Lack L. A brief afternoon nap following nocturnal sleep restriction: which nap duration is most recuperative? Sleep. 2006;29(6):831–840. doi: 10.1093/sleep/29.6.831 16796222

[36] Tassi P , Muzet A. Sleep inertia. Sleep Med Rev. 2000;4(4):341–353. doi: 10.1053/smrv.2000.0098 12531174

[37] Faraut B , Andrillon T, Vecchierini MF, Leger DN. A public health issue. From epidemiological to laboratory studies. Sleep Med Rev. 2017;35:85–100. 27751677 10.1016/j.smrv.2016.09.002

[38] Duggan KA , McDevitt EA, Whitehurst LN, Mednick SC. To nap, perchance to DREAM: a factor analysis of college students’ self-reported reasons for napping. Behav Sleep Med. 2018;16(2):135–153. doi: 10.1080/15402002.2016.1178115 27347727 PMC5374038

[39] Yeo SC , Jos AM, Erwin C, et al. Associations of sleep duration on school nights with self-rated health, overweight, and depression symptoms in adolescents: problems and possible solutions. Sleep Med. 2019;60:96–108. doi: 10.1016/j.sleep.2018.10.041 30611714

[40] Olds T , Blunden S, Petkov J, Forchino F. The relationships between sex, age, geography and time in bed in adolescents: a meta-analysis of data from 23 countries. Sleep Med Rev. 2010;14(6):371–378. doi: 10.1016/j.smrv.2009.12.002 20207558

[41] Marino M , Li Y, Rueschman MN, et al. Measuring sleep: accuracy, sensitivity, and specificity of wrist actigraphy compared to polysomnography. Sleep. 2013;36(11):1747–1755. doi: 10.5665/sleep.3142 24179309 PMC3792393

[42] Ancoli-Israel S , Cole R, Alessi C, Chambers M, Moorcroft W, Pollak CP. The role of actigraphy in the study of sleep and circadian rhythms. Sleep. 2003;26(3):342–392. doi: 10.1093/sleep/26.3.342 12749557

[43] Johnson NL , Kirchner HL, Rosen CL, et al. Sleep estimation using wrist actigraphy in adolescents with and without sleep disordered breathing: a comparison of three data modes. Sleep. 2007;30(7):899–905. doi: 10.1093/sleep/30.7.899 17682661 PMC1978368

[44] Pesonen AK , Kuula L. The validity of a new consumer-targeted wrist device in sleep measurement: an overnight comparison against polysomnography in children and adolescents. J Clin Sleep Med. 2018;14(4):585–591. doi: 10.5664/jcsm.7050 29609722 PMC5886436

[45] Lee XK , Chee N, Ong JL, et al. Validation of a consumer sleep wearable device with actigraphy and polysomnography in adolescents across sleep opportunity manipulations. J Clin Sleep Med. 2019;15(9):1337–1346. 31538605 10.5664/jcsm.7932 PMC6760396

[46] Cellini N , Buman MP, McDevitt EA, Ricker AA, Mednick SC. Direct comparison of two actigraphy devices with polysomnographically recorded naps in healthy young adults. Chronobiol Int. 2013;30(5):691–698. doi: 10.3109/07420528.2013.782312 23721120

[47] Kanady JC , Drummond SP, Mednick SC. Actigraphic assessment of a polysomnographic-recorded nap: a validation study. J Sleep Res. 2011;20(1 Pt 2):214–222. doi: 10.1111/j.1365-2869.2010.00858.x 20626612

