# Supplementary material for: Bidirectional associations between the duration and timing of nocturnal sleep and daytime naps in adolescents differ from weekdays to weekends
Source: Sleep. 2024 Jun 28;47(9):zsae147. doi: 10.1093/sleep/zsae147 (PMC11381561; doi:10.1093/sleep/zsae147)
Supplement: zsae147_suppl_Supplementary_Materials [file zsae147_suppl_supplementary_materials.docx]

Bidirectional associations between the duration and timing of nocturnal sleep and daytime naps in adolescents differ from weekdays to weekends

Ruth L. F. Leong, Ph.D.^1^, Liang Tian^1^, Nicole Yu^1^, Teo Teck Boon^1^, Ju Lynn Ong, Ph.D.^1^, Michael W. L. Chee, M.B.B.S.^1*^

^1^Centre for Sleep and Cognition, Yong Loo Lin School of Medicine, National University of Singapore

*Corresponding authors:

Michael W. L. Chee, Ruth L. F. Leong

Centre for Sleep and Cognition

NUS Yong Loo Lin School of Medicine,

MD1, 12 Science Drive 2

Singapore 117549

E-mails: [michael.chee@nus.edu.sg](mailto:michael.chee@nus.edu.sg), [ruthleong@nus.edu.sg](mailto:ruthleong@nus.edu.sg)

**Supplementary Table 1.** Unstandardized beta (standard error) values for models examining the effects of the previous night’s sleep parameters on the next day’s nap parameters which include the interaction term of weekday/weekend status (WD/WE) and the main sleep predictor for both between and within subjects levels.


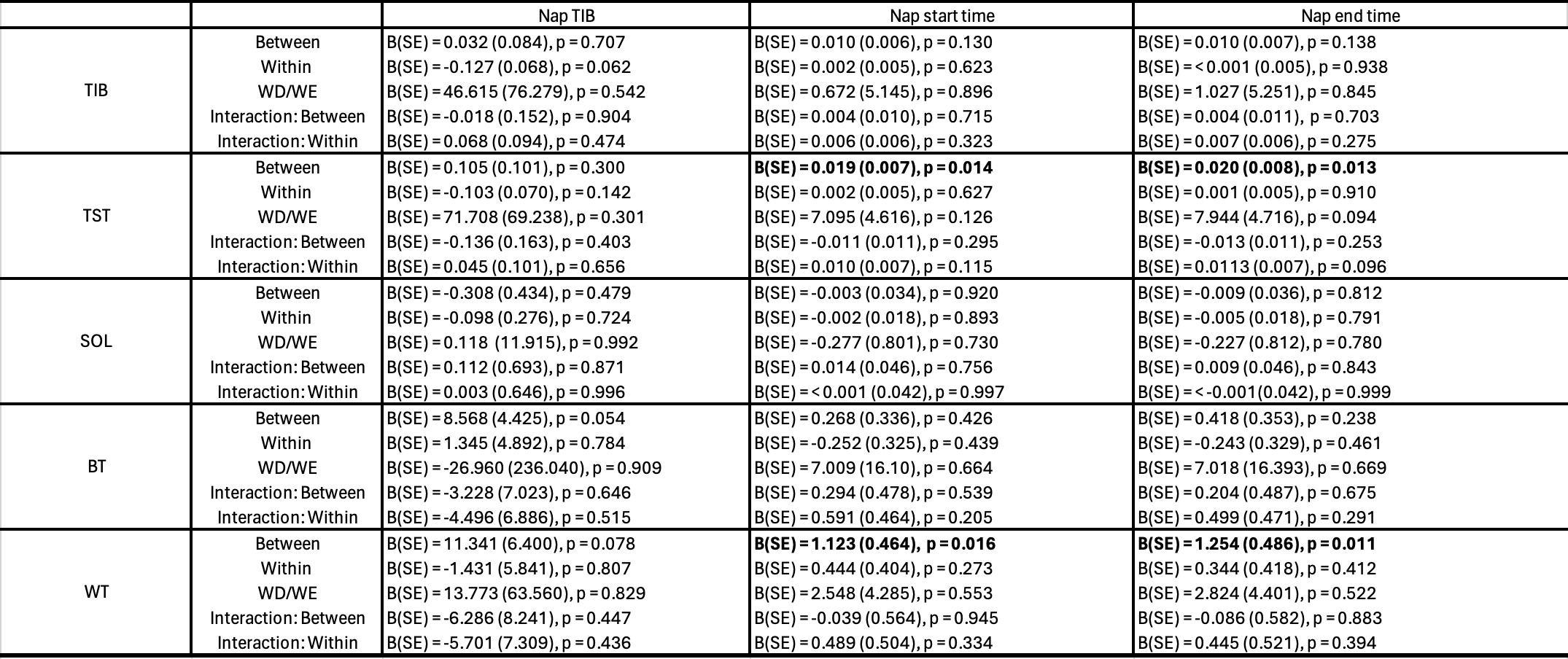


TIB, time in bed; TST, total sleep time; SOL, sleep onset latency; BT, bedtime; WT, wake time

B = Unstandardised beta; SE = standard error.

Bolded values indicate significance at p < 0.05.


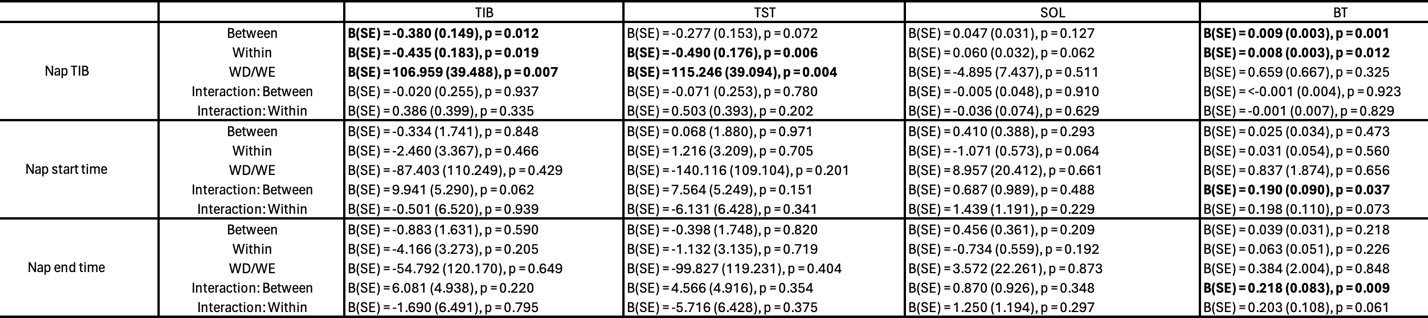
**Supplementary Table 2.** Unstandardized beta (standard error) values for models examining the effects of the same day’s nap parameters on the same night’s sleep which include the interaction term of weekday/weekend status (WD/WE) and the main sleep predictor for both between and within subjects levels.

TIB, time in bed; TST, total sleep time; SOL, sleep onset latency; BT, bedtime

B = Unstandardised beta; SE = standard error.

Bolded values indicate significance at p < 0.05.
